# Predictors for failure of supraglottic superimposed high‐frequency jet ventilation during endoscopic upper airway surgery in pediatric patients

**DOI:** 10.1111/pan.13952

**Published:** 2020-07-17

**Authors:** Charlotte M. A. Plate, Grita Krenz, Bouwe Molenbuur, Frederik G. Dikkers, Geert B. Eindhoven, Jan E. Wachters, Boukje A. C. van Dijk, Gyorgy B. Halmos

**Affiliations:** ^1^ Department of Otorhinolaryngology/Head and Neck Surgery University Medical Center Groningen University of Groningen Groningen The Netherlands; ^2^ Department of Anesthesiology University Medical Center Groningen University of Groningen Groningen The Netherlands; ^3^ Department of Otorhinolaryngology Amsterdam UMC University of Amsterdam Amsterdam The Netherlands; ^4^ Department of Research Netherlands Comprehensive Cancer Organization (IKNL) Utrecht The Netherlands; ^5^ Department of Epidemiology University Medical Center Groningen University of Groningen Groningen The Netherlands

## 1 INTRODUCTION

Airway surgery in pediatric patients is challenging with regard to balancing surgical exposure with ventilation requirements, as during the procedure the airway must be shared between laryngologist and anesthetist. For endoscopic laryngeal surgery, different methods of ventilation are used, among others jet ventilation via a specifically adapted suspension laryngoscope using a dual jet stream (supraglottic superimposed high‐frequency jet ventilation, SSHFJV).^1^ High BMI and a history of pulmonary pathology proved to be factors contributing to failing of SSHFJV in adult patients.^2^ However, factors influencing the failure of SSHFJV in pediatric patients have never been described yet.

## 2 MATERIALS AND METHODS

Retrospectively, a database was analyzed covering 99 surgeries in 65 patients younger than 18 years of age, undergoing upper airway surgery while being ventilated with SSHFJV (device: modified Benjamin‐Lindholm laryngoscope, Carl Reiner GmbH, Austria; ventilator: TwinStream™ Multi Mode Respirator, Carl Reiner GmbH, Vienna, Austria). All patients were treated between 2007 and 2017 at the University Medical Center Groningen, the Netherlands, a tertiary referral hospital. All procedures were performed by dedicated and well‐trained anesthetic and surgical teams. Relevant data from the electronic patient files, including anesthetic reports, surgical reports, and intra‐operative images, were retrospectively analyzed. Baseline characteristics included age and weight at the time of the surgery, gender, laryngological history, diagnosis, treatment, comorbidities, airway anatomy, ASA score, anatomical level, and the severity of stenosis. Patients who underwent upper airway surgery and successfully ventilated with SSHFJV were compared to patients in which SSHFJV was inadequate; in those cases, ventilation had to be temporarily or definitively converted to an alternative ventilation method. The variables were analyzed with IBM SPSS statistics 23 (IBM, Armonk, NY, USA). The two groups were compared using univariable logistic regression, calculating odds ratios with a 95% confidence interval and *P*‐values (Table 1). The Medical Ethics Review Board of our institution reviewed this study and released a waiver.

**TABLE 1:** Univariable analysis, factors leading to both definitive and temporary conversion to an alternative method of ventilation in 99 procedures of 65 pediatric patients (0‐18 y old)

	Odds Ratio (95% CI)	*P*‐value
Age	1 (1.00‐1.00)	.48
Sex		
Male	1	
Female	1.68 (0.51‐5.54)	.40
Weight	0.97 (0.92‐1.02)	.24
Laryngological history		
Positive	1	
Negative	0.70 (0.18‐2.72)	.60
Anatomic level pathology		
Supraglottic	1	.58
Glottic	0.55 (0.06‐4.76)	.15
Subglottic, tracheal	0.21 (0.03‐1.75)	
Diagnosis		
Benign	1	
Laryngomalacia	1.46 (0.40‐5.31)	.56
Stenosis	0.50 (0.05‐4.88)	.55
Treatment		
Intervention on airway stenosis	1	
Supraglottoplasty	2.73 (0.32‐23.65)	.36
Excision benign lesion, other	2.22 (0.23‐21.74)	.49
Cardiovascular comorbidity		
Yes	1	
No	1.16 (0.23‐5.79)	.86
Pulmonary comorbidity		
Yes	1	
No	3.18 (0.82‐12.37)	.10
ASA class		
1, 2	1	
3, 4	2.65 (0.72‐9.83)	.14
Increase of 10% lumen obstruction	1.23 (1.01‐1.51)	**.04**
Mallampati score		
1, 2	1	
3, 4	2.40 (0.34‐16.90)	.38
Surgical factors		
Use of laser		
Yes	1	
No	0.73 (0.19‐2.89)	.66

Note

Statistical test used: univariable logistic regression. Significant *P*‐values are indicated with bold numbers.

Abbreviations: ACE‐27, Adult Comorbidity Evaluation‐27 index; ASA class, American Society of Anesthesiologist Physical Status Classification; BMI, body mass index; CI, confidence interval.

## 3 RESULTS

No complications occurred during the procedures. However, in 13 (13%) cases, ventilation had to be converted to a different ventilation method, predominantly due to desaturation during SSHFJV. Of the converted patients, 9 were temporarily converted and 4 definitively. In all converted cases, patients were under the age of 9 years. In univariable analysis, the only significant factor for (both temporary and definitive) conversion to a different ventilation technique was the percentage of lumen obstruction during the surgery with an odds ratio of 1.23 with an increase in lumen obstruction of 10%, implying an increased chance of failure of 23% with an decrease of 10% lumen. All other variables (eg, age and weight) were not statistically significant.

## 4 DISCUSSION

Based on this retrospective analysis, SSHFJV is applicable in the majority of the pediatric patients. The only factor contributing to failure of SSHFJV in pediatric patients undergoing upper airway surgery is the severity of airway obstruction. If a pediatric patient with a high‐grade stenosis is undergoing an intervention using SSHFJV, pre‐operatively alternative ventilation techniques should be evaluated, prepared, and made ready to use.

Severe comorbidities in pediatric patients with severe stenosis including syndromic abnormalities, severe cardiovascular abnormalities, and neurological impairment have been previously described.^3^ This correlation may contribute to a higher failing rate in patients with higher grade stenosis,however, comorbidities were found not to be related to SSHFJV failure in our series. However, a selection bias probably played a role in our analysis, as multimorbid patients might not have been selected for surgery with SSHFJV. In contrast to the results of the present study, in adults, (severe) stenosis was not a contributing factor to failing of SSHFJV,^2^ emphasizing the remarkable differences between the two patient populations. Furthermore, the airflow during SSHFJV is also highly influenced by the position of the ventilating laryngoscope,laminar flow is desired for optimal ventilation. In high‐grade stenosis, both inspiration and expiration of air can be very limited.

## 5 CONCLUSION

The present study identified the grade of stenosis as the only factor contributing to the failure of this ventilation technique. However, severe airway stenosis is not a contraindication for SSHFJV, but the surgical and anesthetic teams have to be prepared for conversion for alternative ventilation techniques in these cases.
